# KRAS Mutational Regression Is Associated With Oligo-Metastatic Status and Good Prognosis in Metastatic Colorectal Cancer

**DOI:** 10.3389/fonc.2021.632962

**Published:** 2021-03-29

**Authors:** Alessandro Ottaiano, Guglielmo Nasti, Mariachiara Santorsola, Vincenzo Altieri, Giuseppina Di Fruscio, Luisa Circelli, Amalia Luce, Alessia Maria Cossu, Giosuè Scognamiglio, Francesco Perri, Marco Correra, Andrea Belli, Paolo Delrio, Gerardo Botti, Michele Caraglia

**Affiliations:** ^1^ Istituto Nazionale Tumori di Napoli, IRCCS “G. Pascale”, Naples, Italy; ^2^ INNOVALAB, Centro Direzionale Isola A2, Naples, Italy; ^3^ Department of Precision Oncology, AMES-Centro Polidiagnostico Strumentale, Casalnuovo di Napoli, Italy; ^4^ Department of Precision Medicine, University of Campania “L. Vanvitelli”, Naples, Italy; ^5^ Biogem Scarl, Institute of Genetic Research, Laboratory of Precision and Molecular Oncology, Ariano Irpino, Italy

**Keywords:** *KRAS*, metastatic colorectal cancer, DNA, liquid biopsy, prognosis

## Abstract

**Background:**

We previously reported that loss of *KRAS* mutations (“regressive” mutational trajectories) from primary tumors to metastases associated with the oligo-metastatic status in colorectal cancer (CRC). The present study was undertaken in order to analyze the mutational trajectories of *KRAS* in a well-characterized cohort of CRC patients who developed poly- or oligo-metastatic disease.

**Material and Methods:**

Patients were treated and followed-up according to European Society of Medical Oncology guidelines. Primary CRC FFPE tissue and metastatic circulating-free DNA were extracted using the QIAamp DNA specific kits (Qiagen, Hilden, Germany). Samples were sequenced with the Oncomine Solid Tumour DNA kit (Thermo Fisher Scientific, Waltham, MA, USA). Plasma collection for liquid biopsy was done from 1 to 14 days before starting first-line chemotherapy. Analysis of the prognostic power of *KRAS* evolutionary trajectories was done with uni- and multivariate analyses.

**Results:**

One-hundred-fourteen patients were enrolled. Sixty-three patients presented with mutated *KRAS* (mut*KRAS*) and 51 with wild-type *KRAS* (wt*KRAS*). *KRAS* mutational concordance was high (70.1%).Two divergent subsets were identified: mut*KRAS* in primary tumors and wt*KRAS* in metastatic ones (regressive: mut*KRAS* → wt*KRAS* in 8.8% of patients), and *vice versa* (progressive: wtK*RAS* → mut*KRAS* in 21.1% of patients). An association between *KRAS* regressive trajectory and the oligo-metastatic status (P <0.0001) was found. At multivariate analysis, regressive and progressive mutational trajectories emerged as independent prognostic factors for survival, with Hazard Ratios of 0.22 (CI 95%: 0.08–0.61; median survival: not reached) and 2.70 (CI 95%: 1.11–6.56, median survival: 12.1 months), respectively.

**Conclusions:**

Our data provide evidence that the evolutionary trajectories of *KRAS* can have a strong clinical prognostic role and that they can be involved in discriminating between poly-metastatic aggressive *vs* oligo-metastatic indolent CRC.

## Introduction

Colorectal cancer (CRC) is the third cause of cancer-related death worldwide (1). About 30% of patients present at diagnosis with metastatic disease, and half of them will develop metastases after surgical resection of the primary tumor (2). The survival of metastatic colorectal cancer (mCRC) patients significantly improved in the last 20 years with the introduction of target-oriented drugs [anti-EGFR (Epidermal Growth Factor Receptor) and anti-angiogenic agents] associated with chemotherapy (fluoropirimidines, oxaliplatin and irinotecan); however, it still very rarely encompasses 30 months (3). The selection of patients on a genetic basis allowed the selection of those more prone to respond to specific treatments. In fact, it is now clear that mCRC patients bearing specific *KRAS* (Kirsten RAt Sarcoma viral oncogene homolog) mutations do not benefit from anti-EGFR treatment because mutated and constitutively hyper-activated *KRAS* determine a ligand-independent activation of EGFR (4). We previously reported that loss of *KRAS* mutations (“regressive” mutational trajectories) from primary tumors to metastases on FFPE (Formalin-Fixed Paraffin Embedded) resected tissues was associated with long-term survivals and the oligo-metastatic status in mCRC (5, 6). However, the evaluation of circulating tumor DNA (ctDNA) sequences, also called “liquid biopsy”, has provided a great opportunity to study the mutational evolution of cancers with a non-invasive, real-time and repeatable approach. On these bases, Misale et al. (7) demonstrated that the occurrence of *KRAS* point mutations preceded the resistance to anti-EGFR monoclonal antibodies in mCRC patients who experienced an initial response. Furthermore, Siravegna et al. (8) showed that in mCRC patients, during anti-EGFR treatment withdrawals, *KRAS* mutated mCRC cells regain drug-sensitivity due to decay in frequency of *KRAS* mutations which, in some cases, become undetectable. Altogether, these data indicate that mCRC genetics is dynamic and that the evaluation of the tumor mutational status in a single moment could not be representative of the cancer mutational evolution.

The present study was undertaken in order to analyze the mutational trajectories of *KRAS* in a well-characterized cohort of mCRC patients and to correlate those trajectories with the prognosis and the extent of the disease (oligo- *versus* poly-metastatic status).

## Material and Methods

### Patients’ Selection and Management

This was a retrospective, non-interventional and biomarkers study officially approved by the Scientific Directorate on November 11, 2020. The source of data was the electronic database reporting clinical records of CRC patients who underwent to radical excision of primary tumor from 2015 to 2018 and characterization of *KRAS* mutational status. Thereafter, they developed metastatic and unresectable disease and were enrolled into the study upon signature of an informed consent to perform a liquid biopsy for *KRAS* reassessment just before starting the first-line chemotherapy. The treatments were administered at the SSD (*Struttura Semplice Dipartimentale*) Innovative Therapies for Abdominal Metastases of the *Istituto Nazionale Tumori di Napoli, IRCCS “G. Pascale*. Oligo-metastatic patients were intended as those having one to three lesions per organ with a maximum tumor diameter smaller than 70 mm and no lesions encompassing 25 mm diameter. To avoid clear negative prognostic influences, some clinical criteria for patients’ inclusion were established *a priori* and consisted on: Performance Status ECOG (Eastern Cooperative Oncology Group) 0 or 1, age <80 years and life expectancy of at least three months. According to these criteria, 114 patients were selected: 63 had *KRAS* mutations (mutated *KRAS*: mut*KRAS*), 51 were *KRAS* wild-type (wt*KRAS*) (see DNA sequencing). Treatments were chosen according to ESMO (European Society of Medical Oncology) guidelines (9). All patients signed a written informed consent before treatment administration and molecular assessments. The primary outcome of this study was the analysis of the prognostic power of different *KRAS* evolutionary trajectories between the mutational status in primary tumor and that in liquid biopsy at metastases occurrence in both wild-type (wt*KRAS* → wt*KRAS* and wt*KRAS* → mut*KRAS*) and mutated (mut*KRAS* → mut*KRAS* and mut*KRAS* → wt*KRAS*) CRCs. Patients harboring double mutations of *KRAS* or *NRAS* or *BRAF* (v-raf murine sarcoma viral oncogene homolog B) mutations were not included in this study.

### Patients Follow-Up

Total body Computed Tomography (tbCT) scan and CEA (CarcinoEmbryonic Antigen) monitoring were not centralized and were done every three months. The response to chemotherapy was evaluated by RECIST (Response Evaluation Criteria In Solid Tumours v1.1) (10). Complete response (CR) was defined as complete disappearance of all detectable evidence of disease on tbCT. Partial response (PR) was defined as at least a 30% decrease in the sum of diameters of target lesions. Stable disease (SD) was defined as everything between 30% decrease and 20% growth of tumour size. Progressive disease (PD) was defined as at least a 20% increase in the sum of diameters of target lesions. Disease Control (DC) was the sum of CR + PR + SD.

### Plasma Collection

Six mL of whole blood was collected through sting of a peripheral vein, using Vacutainer^®^ with EDTA as anticoagulant (K2EDTA, purple cap, Becton Dickinson). Plasma was separated by two sequential centrifugation steps (the first at room temperature for 10 min at 1,500×*g* and the second at 2,000×*g* for the same time and temperature). Plasma was stored at −80°C until analysis (see beyond).

### Plasma Circulating-Free and Formalin-Fixed Paraffin-Embedded (FFPE) Tissue DNA Extraction

Circulating-free (cf)-DNA was extracted from 1-ml samples of plasma with a commercial kit (QIAamp Circulating Nucleic Acid Kit; QIAGEN GmbH, Hilden, Germany) according to the manufacturer instructions. Cf-DNA samples were then stored at −20°C. FFPE tissue DNA was extracted from three 10 µm FFPE sections using the QIAamp DNA FFPE Tissue kit (Qiagen, Hilden, Germany) and the QIAcube apparatus (Qiagen). The DNA quantity was evaluated with the dsDNA HS assay kit using the Qubit 2.0 Fluorometer (Invitrogen, Monza, Italy).

### DNA Sequencing

Tumour samples were sequenced with the Oncomine Solid Tumour DNA kit (Thermo Fisher Scientific, Waltham, MA, USA) covering hotspot variants and actionable mutations of 22 genes involved in colon cancer. However, our analysis focused on *KRAS*-related genetic results. Ten nanograms of genomic DNA (gDNA) were used to prepare libraries according to the manufacturer’s instructions.

The amplified libraries were sequenced on the Ion Torrent PGM semiconductor (https://www.thermofisher.com/it/en/home/life-science/sequencing/next-generation-sequencing/ion-torrent-next-generation-sequencingworkflow.html) and the data were analyzed using the torrent suite software v5.0 (Thermo Fisher Scientific) and the obtained variants confirmed by the integrative genome viewer (IGV) from the Broad Institute. The limit of mutations detection (LOD) of tissue NGS approach is 2% allelic frequency. Reference sequence for *KRAS* was NM_004958.4. Mutations were also checked according to ClinVar identifier numbers (https://www.ncbi.nlm.nih.gov/clinvar/intro/).

### Statistical Analyses, Study Design, and Data Presentation

Associations between *KRAS* mutations and clinical and pathologic variables were evaluated by χ^2^ test. P <0.05 was considered statistically significant. The primary outcome measure was the Overall Survival (OS), measured from the start of the first-line chemotherapy until death from any cause. The Kaplan–Meier product limit method was applied to graph OS. The study was exploratory considering the scarcity of data about the prognostic power of different mutational evolutions of *KRAS* oncogene in primary *vs* metastatic lesions and, thus, it does not have a pre-specified study design. All patients registered in an observational database (STORIA database) (11) between 2015 and 2018 and who accepted to perform liquid biopsy before starting first-line chemotherapy were enrolled. We chose do not prolong the enrolment period to avoid any prognostic interferences related to therapeutic and methodologic changes occurring in clinical practice. With an estimated survival difference between patients with *KRAS* mutational regression (defined as an expected rare group, mut*KRAS* → wt*KRAS*) *vs* patients with stably mutated *KRAS* (mut*KRAS* → mut*KRAS*) higher than 50% at 12 months, an estimated ratio mut*KRAS* → wt*KRAS*:mut*KRAS* → mut*KRAS* of about 1:10, a sample size of at least 60 patients was required to generate a significant hypothesis (*P <*0.005) on survival time differences at Log-Rank Test.

Univariate analysis was performed with the Log-Rank test. Multivariate analysis was performed through the Cox proportional-hazards regression in order to analyze the effect of several risk factors (co-variates) on OS. The HR is the estimate of the end-point probability and it can be interpreted as the instantaneous relative risk of an event (death), at any time, for an individual with the risk factor present compared with an individual with the risk factor absent, given both individuals are the same on all other covariates. Covariates were selected after consensus discussion between authors and were dichotomized: age <65 *vs* age ≥65, male *vs* female, left sided *vs* right sided, one involved organ *vs* two or more, response to first-line chemotherapy (Disease Control *vs* No Disease Control), *KRAS* mutational evolution in mutated KRAS (mut*KRAS* → mut*KRAS vs* mut*KRAS* → wt*KRAS*) and in wild-type KRAS (wt*KRAS* → wt*KRAS vs* wt*KRAS* → mut*KRAS*). 95% confidence intervals (CI) of HR are also reported. Statistical analysis was performed using the MedCalc^®^ 9.3.7.0 and Excel software.

## Results

### Clinico-Pathological and Treatment Characteristics According to the Initial Mutational Status of KRAS

One-hundred-fourteen patients who received surgical removal and *KRAS* oncogene evaluation of a primary CRC between 2015 and 2018 accepted to reassess *KRAS* mutational status through liquid biopsy before starting the first-line chemotherapy for the occurrence of distant and non-resectable metastases. However, first-line and subsequent chemotherapies were established according to the *KRAS* assessed on the primary FFPE tumoral tissue as established by National Regulatory Authorities (a detailed description is reported in **Table S1**). **Table 1** shows the clinic-pathologic characteristics of patients according to the *KRAS* mutational status in primary tumors. Overall, 63 patients presented with mut*KRAS* and 51 with wt*KRAS*. The three most frequent mutations were p.G12D (19 patients), p.G13D (nine patients) and p.G12V (seven patients). There were no statistically significant associations at χ^2^ test between the mutational status of *KRAS* on primary tumors and age (<65 *vs* ≥65 years), gender (male *vs* female), grading (G1/G2 *vs* G3), side of primary tumor (left *vs* right), pT (pT1/pT2 *vs* pT3 *vs* pT4), and lymphnodes involvement (pN: 0 *vs* 1–3 *vs >*3). According to Oncology Societies’ guidelines and National Pharmaceutical Authorities’ regulations, patients bearing mut*KRAS* in primary tumors did not receive anti-EGFR-based treatments (**Table 2**). In this patients’ setting, the use of chemotherapy (CT) and bevacizumab was predominant (56/63 patients, 88.8%); conversely, in wt*KRAS* patients the 76.4% of them was treated with CT plus an anti-EGFR agent (39/51 patients). Interestingly, wt*KRAS* patients received more CT lines (43.3% *vs* 20.6% in mut*KRAS* patients) and had a longer cumulative median time-on-therapy (20.5 *vs* 16.9 months in mut*KRAS* patients). This was indirectly related to the detrimental prognostic effect on survival of mut*KRAS* (11).

**Table 1:** Clinico-pathological characteristics according to KRAS status at diagnosis.

KRAS status in primary tumor	Total	Age		Gender		Grading		Side of primary tumor		pT*			Lymph nodes involvement (pN)*		
		<65	≥65	M	F	G1/G2	G3	Left	Right	T1/T2	T3	T4	0	1–3	>3
**p.G12D**	**63**	7	12	11	8	5	14	8	11	3	9	7	2	5	12
**p.G13D**		5	4	3	6	1	8	4	5	1	7	1	1	7	1
**p.G12V**		3	4	3	4	1	6	2	5	1	5	1	0	5	2
**p.G12A**		3	3	3	3	1	5	2	4	1	4	1	0	3	3
**p.G12C**		4	2	3	3	0	6	1	5	2	3	1	1	3	2
**p.A146T**		3	2	3	2	1	4	2	3	0	3	2	0	2	3
**p.G12S**		1	3	2	2	0	4	0	4	1	2	1	1	3	0
**p.A146V**		1	1	1	1	0	2	0	2	1	1	0	0	2	0
**p.G13R**		0	2	0	2	0	2	1	1	2	0	0	1	0	1
**p.G13C**		0	1	1	0	1	0	1	0	0	1	1	0	1	1
**p.K117N**		1	0	0	1	0	1	0	1	0	0	1	1	0	0
**p.G12F**		1	0	1	0	0	1	1	0	1	0	0	1	0	0
**Wild-type**	**51**	18	23	20	21	9	32	28	23	11	21	9	15	19	17

pT, pathological staging of primary tumor according to AJCC; pN, pathological staging of loco-regional lymph-nodes involvement. ^*^According to AJCC. There were no significant associations between KRAS mutations and clinical and pathologic variables at χ^2^ test. Sequence change is described at protein level with "p." followed by the amino acid abbreviation, followed by the position of the amino acid sequence, followed by the new amino acid which replaces the former.

**Table 2:** Treatment characteristics according to KRAS status at diagnosis.

KRAS status in primary tumor	Total	Type of first-line CT			No. of CT lines			Time on therapy*(months)
		CT	CT/Beva	CT/anti-EGFR	1	2	>2	Median (Range)
**p.G12D**	**63**	2	17	0	7	8	4	15.9 (12.3–21.5)**
**p.G13D**		1	8	0	3	4	2	
**p.G12V**		1	6	0	1	2	4	
**p.G12A**		0	6	0	1	5	0	
**p.G12C**		1	5	0	4	2	0	
**p.A146T**		0	5	0	1	4	0	
**p.G12S**		0	4	0	2	1	1	
**p.A146V**		1	1	0	0	1	1	
**p.G13R**		0	2	0	0	2	0	
**p.G13C**		0	1	0	0	0	1	
**p.K117N**		0	1	0	0	1	0	
**p.G12F**		1	0	0	0	1	0	
**Wild-type**	**51**	8	4	39	8	20	23	20.6 (16.2–27.6)

anti-EGFR, Anti-Epidermal Growth Factor Receptor antibodies; Beva, Bevacizumab; CT, ChemoTherapy.

*Cumulative time spent on therapy (including also “maintenance therapy”).

**Time-on-therapy for all mutated patients. Sequence change is described at protein level with "p." followed by the amino acid abbreviation, followed by the position of the amino acid sequence, followed by the new amino acid which replaces the former.

### Genetic Evolution of KRAS

KRAS mutational concordance (mut*KRAS* or wt*KRAS* in both primary and liquid biopsy at metastases occurrence: mut*KRAS* → mut*KRAS* and wt*KRAS* → wt*KRAS*) was high (70.1%). However, two divergent subsets were identified: 1. mut*KRAS* in primary tumors and wt*KRAS* in metastatic ones (mut*KRAS* → wt*KRAS* in 8.8% of patients), and *vice versa* (wt*KRAS* → mut*KRAS* in 21.1% of patients) (**Table 3**). These subsets are particularly interesting because they represent a dynamic aspect of cancer heterogeneity. **Table 3** shows some clinical characteristics that could have influenced the genetic evolution of *KRAS*. There were statistically significant associations: 1. adjuvant chemotherapy based on capecitabine and oxaliplatin more frequently preceded the evolution towards mut*KRAS* from wt*KRAS* (wt*KRAS* → mut*KRAS*) (P = 0.001), 2. There was a strong association between *KRAS* regressive trajectory (mut*KRAS* → wt*KRAS*) and the oligometastatic status (P <0.0001) (see *Material and Methods* for the definition of oligo-metastatic disease), 3. Regression of mut*KRAS* (mut*KRAS* → wt*KRAS*) before starting first-line chemotherapy was associated with response to CT alone or CT plus bevacizumab (P = 0.026) (**Table 3**).

**Table 3:** Tumor burden, adjuvant chemotherapy and response to first-line CT according to KRAS evolution.

KRAS evolution		Adjuvant CT		P*	Sites of first recurrence				P*	Oligo-metastases		P*	Best response tofirst-line CT			P*
		Yes(52)	No(62)		Liver	Lungs	Lymph-nodes	More thanone site		Yes	No		CR, PR or SD	PD	NA	
Mut in PT → Mut in MT	53	24	29		16	8	7	22		2	51		31	19	3	
Mut in PT → WT in MT	10	3	7		5	4	0	1		9	1		10	0	0	
WT in PT → WT in MT	27	7	20		8	8	4	7		4	23		19	5	1	
WT in PT → Mut in MT	24	19	5	*0.001*	5	3	2	14	*0.058*	1	23	*<0.0001*	11	10	2	*0.026*

CR, Complete Response; CT, ChemoTherapy; MT, Metastatic Tumors; Mut, KRAS mutated; NA, Not Assessable; PD, Progressive Disease; PR, Partial Response; PT, Primary Tumors; SD, Stable Disease; WT, Wild-Type. *Chi-square test.

### Prognostic Significance of KRAS Mutations Evolution

**Figure 1:**
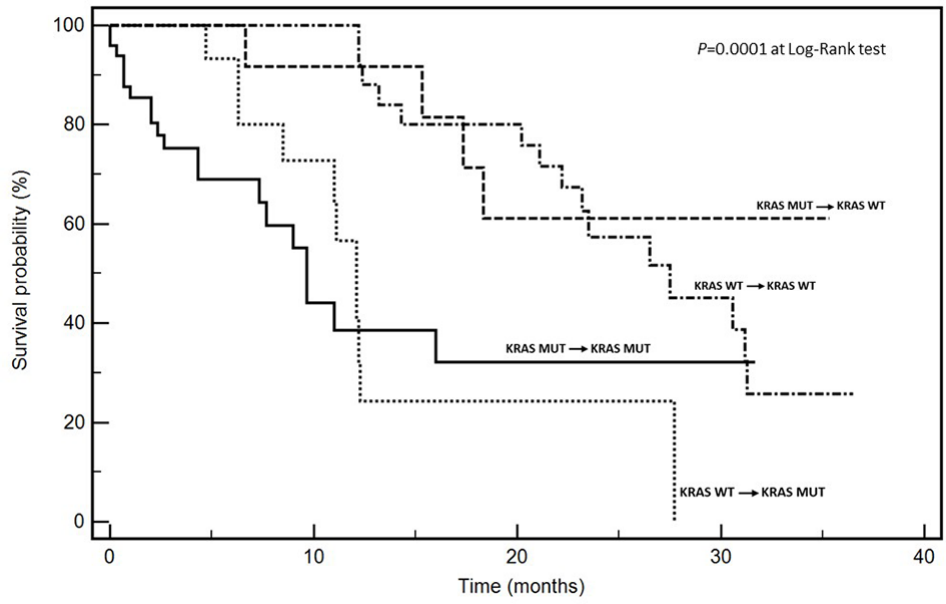
Kaplan-Meier survival curves according to KRAS mutational trajectories.

Given the opportunity to distinguish four *KRAS* evolutionary subsets, we studied the prognostic impact of these subsets on survival, the most reliable and synthetic outcome. Time-to-progression was not evaluated considering the potential prognostic biases related to different first-line chemotherapies and/or different therapeutic sequences. **Table 4** and **Figure 1** show respectively, univariate analysis of overall survival (OS) and Kaplan Meyer curves depicting the survival of patients according to different evolutionary subsets. After a median follow-up for the whole series of 25.0 months, median OS (mOS) of *KRAS* genetically concordant patients was 9.6 for mut*KRAS* → mut *KRAS* and 27.5 months for wt*KRAS* → wt*KRAS*. Median OS (mOS) for mut*KRAS* → wt*KRAS* (“regressive trajectory”) was not reached (NR), while median mOS in patients developing *KRAS* mutations in metastatic tumors from wt*KRAS* in primary lesions (wt*KRAS* → mut*KRAS*) was 12.1 months (P = 0.0001 at Long Rank test).

**Table 4:** Univariate analysis of KRAS mutations’ evolution prognostic power.

KRAS evolution	No. ofevents/patients	Median survival	95% CI	*P* at Log Rank test
Mut in PT → Mut in MT	20/53	9.6	6.7–16.4	
Mut in PT → WT in MT	4/10	NR	21.1–33.6	
WT in PT → WT in MT	15/27	27.5	22.8–29.8	
WT in PT → Mut in MT	11/24	12.1	9.6–15.9	*0.0001*

CI, Confidence Interval; MT, Metastatic Tumors; Mut, KRAS mutated; NR, Not Reached; PT, Primary Tumors; WT, Wild-Type.

A multivariate analysis was performed including as dichotomized co-variates, age (<65 *vs* ≥65 years), gender (male *vs* female), side (left *vs* right), extent of metastatic involvement (one *vs* multiple sites), response to first-line CT [CR/PR/SD (DC, Disease Control) *vs* PD (no DC)], genetic concordance in mut*KRAS vs* regressive trajectory (mut*KRAS* → wt*KRAS*), and genetic concordance in wt*KRAS vs* progressive trajectory (wt*KRAS* → mut*KRAS*). Interestingly, the following conclusions from the statistical analysis can be derived: i. metastatic involvement (one *vs* multiple sites; mOS: 30.6 *vs* 11.0 months; HR: 4.16, CI 1.25–13.7), ii. response to first-line CT (DC *vs* no DC; mOS: 28.3 *vs* 9.6.0 months; HR: 2.11, CI 1.78–4.26), and *KRAS* evolutionary iii. regressive (mut*KRAS* → mut*KRAS vs* mut*KRAS* → wt*KRAS*; mOS: 9.6 months *vs* NR; HR: 0.22, CI 0.08–0.61) and iv. progressive trajectories (wt*KRAS* → wt*KRAS vs* wt*KRAS* → mut*KRAS*; mOS: 27.5 *vs* 12.1 months; HR: 2.70, CI 1.11–6.56) emerged as independent prognostic factors for OS (**Table 5**).

**Table 5:** Multivariate analysis of RAS mutations’ evolution prognostic power.

Co-variate	Dicothomization	Median survivals	No. ofevents/patients	*P* at univariate	HR	95% CI	*P* at multivariate
**Age**	<65 y *vs* ≥65 y	15.3 vs 18.3	12/47 vs 13/57	0.90	0.69	0.24–1.96	0.49
**Gender**	M *vs* F	15.3 vs 17.3	13/51 vs 12/53	0.92	1.05	0.37–2.97	0.91
**Side**	L *vs* R	17.5 vs 16.0	21/50 vs 29/64	0.63	1.57	0.48–5.12	0.44
**Metastatic involvment**	1 site *vs >*1	30.6 vs 11.0	33/70 vs 17/44	0.0006	4.16	1.25–13.7	0.001
**Response to firs-line CT**	DC *vs* no DC	28.3 vs 9.6	22/71 vs 28/43	0.002	2.11	1.78–4.26	0.03
**KRAS evolution**	Mut in PT → Mut in MT vs Mut in PT → WT in MT	9.6 vs NR	20/53 vs 4/10	<0.0001	0.22	0.08–0.61	0.0001
	WT in PT → WT in MT vs WT in PT → Mut in MT	27.5 vs 12.1	15/27 vs 11/24	0.0001	2.70	1.11–6.56	0.002

CI, Confidence Interval; DC, Disease Control; F, Female; HR, Hazard Ratio; L, Left; M, Male; MT, Metastatic Tumors; PT, Primary Tumors; mut, KRAS mutated; NR, Not Reached; R, Right; WT, Wild-Type.

## Discussion

In this work, we found that the genetic dynamics of mCRC is clinically relevant since patients bearing divergent mutational evolution have a prognosis consistent with the results of liquid biopsy: in fact, patients bearing mut*KRAS* at liquid biopsy from a wt*KRAS* in primary tumor have both poorer survival and responsiveness to chemotherapy similar to *KRAS* mutated CRCs. This subset could represent a particularly aggressive phenotype on an evolutionary point of view (“progressive” genetic trajectory). By contrast, in 8.8% of cases we observed a “regressive” mutational trajectory that was associated to the best prognosis and high responsiveness to chemotherapy independently from the lack of anti-EGFR treatment administration. The last data are particularly surprising and could indicate that additional unexplored anti-tumoral mechanisms could work to downsize the neoplastic population. Multivariate analysis showed that both mutational trajectories had an independent and significant prognostic power. As in our previous studies (5, 6), we cannot definitively demonstrate if this effect depends on a negative immunologic selection (12) or on a spontaneous genetic devolution. Our translational studies are in progress to identify and isolate, from oligo-metastatic CRC patients, eventual T-cells responsible of mut*KRAS* clones’ elimination.

Surprisingly, we found that “progressive” genetic trajectories (wt*KRAS* → mut*KRAS*) were much more frequent in patients treated with adjuvant chemotherapy (**Table 3**). These findings were consistent with two previous studies performed in well-defined models of oligo-metastatic CRC (5, 6). We hypothesized that chemotherapy would induce a genetic remodelling. The existence of this selection mechanisms was supported by our findings on much more divergent mutational signatures and events between primary and matched metastases when the resection of metachronous metastases was preceded by adjuvant chemotherapy (mutational sharing: >90% in non-chemotherapy-pre-treated lesions *vs <*15% in chemotherapy-pre-treated lesions). In other words, *RAS* wild-type CRC patients progressing after oxaliplatin/capecitabine-based adjuvant chemotherapy developed more *RAS* mutations and resistance to anti-EGFR treatments than metastatic patients who did not receive adjuvant treatment. Therefore, chemotherapy could induce both genetic remodelling and evolutionary pushing. The neoplastic progeny of chemotherapy pre-treated CRC patients could have much more extensive intra-tumour mutation heterogeneity including some clones evolved towards mut*KRAS*. In our opinion, a similar effectwas observed in a very recent study by Wu et al. (13) reporting a trial on the use of osimertinib in completely resected EGFR mutated non-small cell lung cancers (NSCLC). Osimertinib adjuvant administration was less effective on disease-free survival when preceded by chemotherapy. Importantly, we are describing our scientific observations and not deploring adjuvant chemotherapy that is a standard of care in high-risk resected CRC and NSCLC. A useful suggestion rising from our work would be to reassess the mutational status of *KRAS*, particularly in patients underwent to adjuvant chemotherapies, in order to predict the major risk to develop a chemotherapy-induced genetic remodelling requiring both more aggressive treatment strategies and more careful follow-up.

Some limitations of the present study deserve to be discussed. The sample size is limited to 114 patients. In fact, about half of patients did not accept to reassess *KRAS* with liquid biopsy in order to modulate the planningof the therapeutic approach that remained based on the first FFPE tissue *RAS* evaluation. Furthermore, only 10 patients showed a regressive trajectory of *KRAS* (mut*KRAS* → wt*KRAS*) and this was related to the lower incidence of this effect if compared to the progressive trajectory (wt*KRAS* → mut*KRAS*) which represents an advantageous gain for cancer cells. First-line treatments were physiologically heterogeneous according to clinical and genetic assessments; however, the mono-institutional and exploratory nature of this study, along with the uniformity of technical approaches, makes our results precious and informative. Moreover, we did not evaluate *KRAS* status at different time-points because of budget limitations. This is a limit and a missed opportunity to observe the complete *KRAS* cancer cells “plasticity” during the time. Finally, we did not investigate if wt*KRAS* patients on liquid biopsy were again responsive to anti-EGFR treatments. The latter is a crucial question, which deserves to be explored in large prospective trials. At this stage, we can observe that our patients bearing a regressive trajectory (mut*KRAS* → wt*KRAS*) were responsive to treatments and had a good OS regardless of whether they did not receive anti-EGFR agents in first or second lines of treatments.

This work may have a strong practice-changing power in our context since results of liquid biopsy are considered not standard and the National Sanitary System does not reimburse the relative costs. Our results strongly suggest that a single *KRAS* mutational status determination at the diagnosis is nor correct neither useful because cancer clonal heterogeneity can determine a change of the mutational status over the space (in different sites of disease localization) and time, as already suggested by other researchers (4, 7, 8). In our opinion, our work could contribute to provide a biological basis to approach *KRAS* testing with a more dynamic attitude (liquid biopsy) giving both new prognostic and therapeutic chances. The identification of regressive genetic trajectories (mut*KRAS* → wt*KRAS*) in specific mCRC patients could open unexpected therapeutic scenarios. In fact, in this subset, treatment with anti-EGFR-based drugs (cetuximab or panitumumab) could regain relevance and it deserves to be further explored in clinical trials. Furthermore, our data provides an additional direct evidence that studies of the evolutionary trajectories of *KRAS* can have a strong clinical and prognostic impact also in discriminating between poly-metastatic aggressive *vs* oligo-metastatic indolent CRC subsets.

## Data Availability Statement

The original contributions presented in the study are included in the article/**Supplementary Material**. Further inquiries can be directed to the corresponding author.

## Ethics Statement

Ethical review and approval was not required for the study on human participants in accordance with the local legislation and institutional requirements. The patients/participants provided their written informed consent to participate in this study.

## Author Contributions

Conceptualization, AO, GN, and MS. Methodology, VA, GD, and LC. Software, FP and AB. Validation, GB, GN, and GS. Formal analysis, AO, MaC, and PD. Investigation, AO, GN, AL, and MiC. Resources, GN. Data curation, AO and MiC. Writing/original draft preparation, AO, MS, and MiC. Writing/review and editing, GN, GB, and GS. Supervision, MiC. All authors contributed to the article and approved the submitted version.

## Funding

This work was funded by Mrs. Antonietta Nacca.

## Conflict of Interest

The authors declare that the research was conducted in the absence of any commercial or financial relationships that could be construed as a potential conflict of interest.
