# Supplementary material for: KRAS Mutational Regression Is Associated With Oligo-Metastatic Status and Good Prognosis in Metastatic Colorectal Cancer
Source: Front Oncol. 2021 Mar 29;11:632962. doi: 10.3389/fonc.2021.632962 (PMC8039443; doi:10.3389/fonc.2021.632962)
Supplement: Supplementary file 1 [file Table_1.docx]

**Table S1.** Detailed first-line chemotherapies according to KRAS evolution.

|  | ***Type of chemotherapy*** | | | | | | | | | |
| --- | --- | --- | --- | --- | --- | --- | --- | --- | --- | --- |
| **KRAS evolution** | ***Folfox*** | ***Cape/Oxa*** | ***Folfiri*** | ***Folfox +Beva*** | ***Cape/Oxa +Beva*** | ***Folfiri +Beva*** | ***Folfox +Pani*** | ***Folfox +Cet*** | ***Folfiri +Pani*** | ***Folfiri +Cet*** |
| Mut in PT→Mut in MT | 3 | 3 | 0 | 24 | 6 | 17 | 0 | 0 | 0 | 0 |
| Mut in PT→WT in MT | 0 | 1 | 0 | 4 | 2 | 3 | 0 | 0 | 0 | 0 |
| WT in PT→WT in MT | 3 | 1 | 1 | 1 | 0 | 1 | 13 | 3 | 3 | 1 |
| WT in PT→Mut in MT | *0* | *2* | *1* | *0* | *2* | *0* | *12* | *5* | *2* | *0* |

Folfox: fluorouracil 400 mg intravenous (iv) bolus on day 1, leucovorin 400 mg/mq iv on day 1 over two hours, oxaliplatin 85 mg/mq iv on day 1, fluorouracil 2400 mg/mq iv continuous infusion over 46 hours on day 1, every 14 days.

Cape/Oxa: Capecitabine 1000 mg/mq twice daily *per os* on days 1-14, oxaliplatin 130 mg/mq iv day 1, every three weeks.

Folfiri: fluorouracil 400 mg iv bolus on day 1, leucovorin 400 mg/mq on day 1 iv over two hours, irinotecan 180 mg/mq iv on day 1, fluorouracil 2400 mg/mq iv continuous infusion over 46 hours on day 1, every 14 days.

Beva: Bevacizumab 5 g/kg iv on day 1 with folfox or folfiri, 7.5 mg/kg iv on day 1 every three weeks with Cape/Oxa.

Pani: Panitumumab 6 mg/kg iv on day 1 with folfox or folfiri every two weeks.

Cet: Cetuximab 400 mg/mq iv as initial dose followed by 250 mg/mq weekly with folfox or folfiri.
